# Targeting LSD2 in breast cancer

**DOI:** 10.18632/aging.101371

**Published:** 2018-01-20

**Authors:** Yi Huang, Yongmei Yin, Min Sun

**Affiliations:** 1 Women’s Cancer Research Center, UPMC Hillman Cancer Center, University of Pittsburgh School of Medicine, Pittsburgh, PA15213, USA; 2 Department of Pharmacology & Chemical Biology, University of Pittsburgh School of Medicine, Pittsburgh, PA15213, USA; 3 Department of Oncology, the First Affiliated Hospital of Nanjing Medical University, Nanjing, P.R. China; 4 UPMC St. Margaret Hospital, Pittsburgh, PA15215, USA

**Keywords:** LSD2, breast cancer, LSD1, cancer stem cell, tumorigenesis, small molecule inhibitor, target therapy

**Figure 1:**
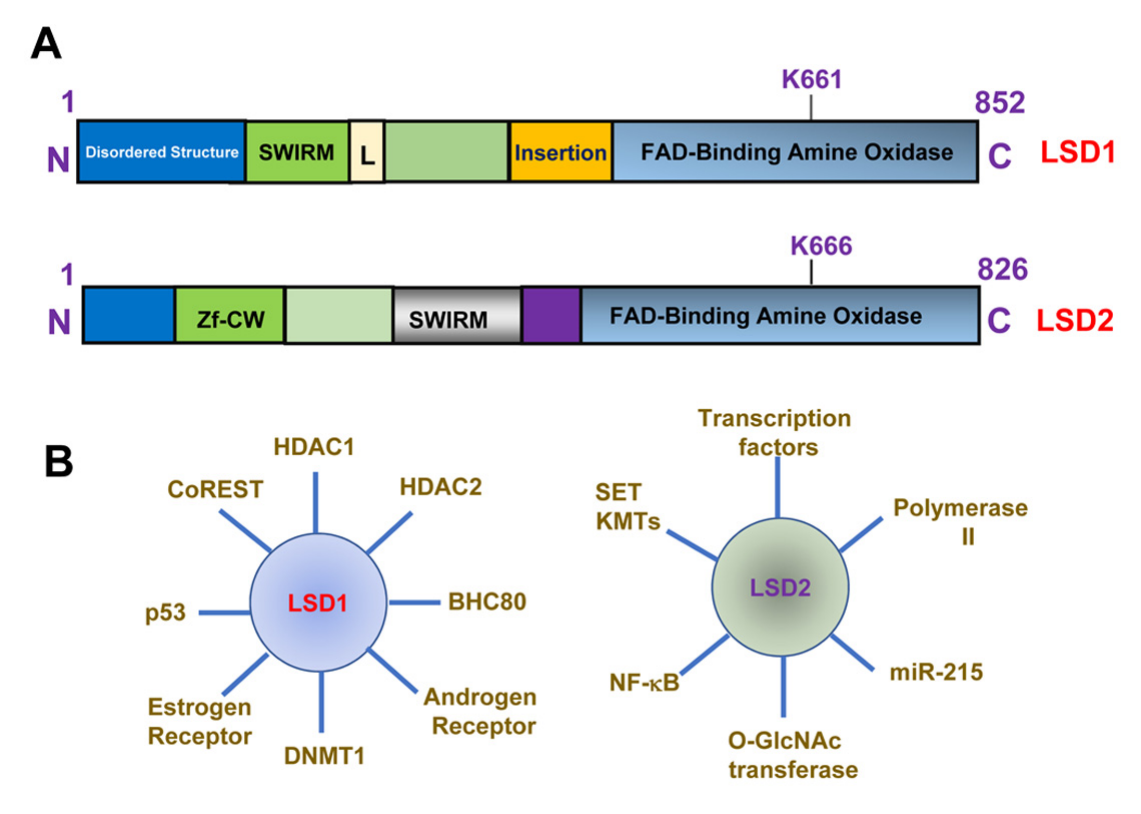
**Structure and function of LSD1 and LSD2.** (**A**) Structure and domain organization of LSD1 and LSD2 proteins. Lysine 661 (K661) and lysine 666 (K666) are FAD binding sites for LSD1 and LSD2. Zf-CW, zinc finger CW domain. SWIRM, Swi3p, Rsc8p and Moira domain. (**B**) Factors that interact with LSD1 or LSD2.

Histone lysine demethylases (KDMs) are family of enzymes which are involved in the extensive epigenetic modification in many cellular processes such as transcription regulation, chromatin remodeling, DNA proofreading and repair, cellular proliferation, embryotic development, etc [1,2]. To date, two families of histone demethylases (KDMs) have been discovered; the flavin-dependent lysine specific demethylases and the JmjC-domain containing KDMs. The flavin-dependent KDM family includes LSD1 (KDM1A/AOF2) and LSD2 (KDM1B/AOF1). Both enzymes contain a SWIRM domain and an amine oxidase (AO) domain. Structurally, LSD1 contains a coiled-coil tower domain protruding from the AO domain responsible for interaction with its co-factors, while LSD2 possesses an aminoterminal zinc finger element that is necessary for LSD2 binding to its methylated substrate. Both enzymes catalyze histone demethylation through a flavin-dependent oxidative process that generates a demethylated lysine 4 of histone 3 and H_2_O_2_ as a byproduct. Although LSD1 and LSD2 share significant homology of amino acid sequence in the AO domain and both enzymes demethylate lysine 4 on histone 3 in a FAD-dependent manner, it is apparent that the two enzymes also have distinct functions, and therefore may act differently in regulation of gene transcription and chromatin remodeling (Figure 1). While LSD1 mostly binds to the promoter region of genes, LSD2 associates primarily with the body regions of actively transcribed genes. LSD1 typically participates in transcriptional repression as part of a protein complex that contains multiple transcriptional corepressors including HDAC1/2, CoREST, BHC80, etc. The dysregulated transcriptional repressive activity of LSD1 complex has been implicated in cancer initiation and progression [3,4]. While the activities of LSD1 in promoting breast cancer progression have been well recognized, the functions of LSD2 in breast tumorigenesis are relatively less characterized.

In our recent published research article, we reported that the level of LSD2 expression is significantly increased in breast cancer specimens in comparison to normal adjacent tissue, and LSD2 expression in invasive breast tumors is greater than that of non-invasive counterparts [5]. These findings indicate a tumor promoting role of LSD2 in breast cancer biology. Our findings also have revealed for the first time that LSD2 overexpression confers cancer stem-cell (CSC) like traits to breast cancer cells. These results indicate that, analogous to LSD1, LSD2 is also required to maintain CSC-like features in breast cancer. However, it is not clear whether LSD2 exerts an additional layer of effect in promoting breast cancer stem cell features through coordinated interaction with LSD1 to alter the methylation level of lysine 4 of histone 3 at CSC regulatory genes. Future work using advanced approaches such as genome-wide mapping and proteomic studies would aid in clarifying the role of crosstalk between FAD-dependent histone demethylase family enzymes in governing biological processes of stem cell development in breast tumors.

LSD2-mediated H3K4 demethylation is involved in establishing the DNA methylation imprints during oogenesis [6]. However, the roles and precise mechanisms of LSD2 in governing DNA methylation are not fully understood. Our lab recently reported that knockdown of LSD2 expression attenuates global DNA methylation, and combined inhibition of LSD2 and DNA methyltransferase (DNMTs) derepressed heavily methylated and silenced tumor suppressor genes, such as *SFRP1, SFRP2* and *CDH1*, that in turn enhanced apoptosis in breast cancer cells [7]. Abnormal suppression of these tumor suppressor genes by LSD2 has been implicated in breast tumor progression and drug resistance. Our findings also suggest that inhibition of LSD2 function could effectively improve the antitumor activity of DNMT inhibitors in breast cancer cells. The precise mechanisms by which LSD2 regulates DNA methylation in breast cancer cells remain unknown. We found that LSD2 overexpression resulted in elevated expression of DNMT3B, which is a critical epigenetic modifier in promoting DNA methylation and gene silencing in cancer [5]. Based on these findings, we speculate that overexpression of LSD2 in breast cancer cells reduces levels of H3K4me2 that could subsequently generate a favorable chromatin environment for recruitment of DNMTs to specific genes to repress their transcriptional activity. Further investigation using related preclinical models is necessary to determine whether combinatorial therapy targeting both LSD2 and DNMTs would be more effective than single agent treatment in breast cancer.

Our studies suggest that LSD2 can serve as a potential therapeutic target for breast cancer treatment. Over the past decade, many promising small molecule inhibitors targeting histone demethylases have been identified or synthesized. The development of novel FAD-dependent KDM inhibitors is progressing rapidly and several clinical trials of LSD1 inhibitors are ongoing in cancer patients such as lung cancer and acute myeloid leukemia. However, some of the LSD1 inhibitors may also affect activity of LSD2 due to the similarities in their catalytic domains, and LSD2-specific inhibitors are as yet unavailable. Since LSD2 is part of transcriptional machinery and chromatin-remodeling complexes that are distinct from those involving LSD1, it is likely to identify compounds that could effectively target LSD2 alone. In the future, it is anticipated that further elucidation of the structure and function of LSD2 complex would facilitate development of more specific and effective LSD2 inhibitors for breast cancer therapy.
